# Successful treatment of critical coronavirus disease 2019 in a patient with lung cancer concomitant with pembrolizumab‐induced arthritis by methylprednisolone, baricitinib, and remdesivir

**DOI:** 10.1002/ccr3.4459

**Published:** 2021-07-06

**Authors:** Naohiro Oda, Keiji Miyoshi, Daisuke Morichika, Yuka Beika, Takahiro Taki, Reo Mitani, Toshiaki Okada, Ichiro Takata

**Affiliations:** ^1^ Department of Internal Medicine Fukuyama City Hospital Fukuyama Japan; ^2^ Department of Respiratory Medicine Fukuyama Medical Center Fukuyama Japan

**Keywords:** baricitinib, COVID‐19, immune checkpoint inhibitor, immune‐related adverse event, lung cancer

## Abstract

COVID‐19 in cancer patients on immunosuppressive agents for the treatment of immune‐related adverse events of immune checkpoint inhibitors can rapidly deteriorate. The combination therapy with methylprednisolone, baricitinib, and remdesivir may be effective for critical COVID‐19, and further clinical trials are warranted.

## 1 INTRODUCTION

Cancer patients have been reported to have a more than twice higher risk of death from coronavirus disease 2019 (COVID‐19) than healthy individuals.^1^, ^2^ Lung cancer has been reported to be the second cancer type with the highest risk of severe COVID‐19 after hematologic malignancies.^2^ In an observational study of thoracic cancer patients (mostly metastatic nonsmall cell lung cancer [NSCLC] patients) with COVID‐19, the following were associated with an increased risk of death: aged >65 years, a current or former smoker, having received chemotherapy treatment within 3 months, and the presence of any comorbidities; conversely, having received treatment with tyrosine kinase inhibitor (TKI) or immune checkpoint inhibitor (ICI) was not associated with an increased risk of death.^3^ However, it is uncertain if patients on immunosuppressive agents for the treatment of immune‐related adverse events (irAEs) are at a greater risk of severe COVID‐19.

Here, we report a case of critical COVID‐19 in a patient with NSCLC concomitant with refractory pembrolizumab‐induced arthritis who was successfully treated with methylprednisolone, baricitinib, and remdesivir.

## 2 CASE REPORT

**FIGURE 1:**
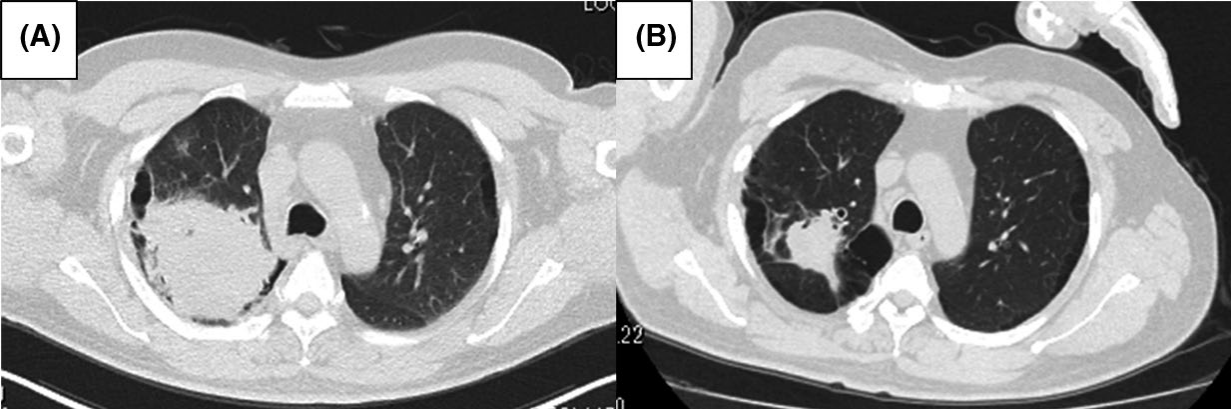
Chest computed tomography showing the primary lesion in the right upper lobe at the time of diagnosis of lung cancer (A) and after four cycles of carboplatin, nab‐paclitaxel, and pembrolizumab and four cycles of pembrolizumab maintenance (B)

A 50‐year‐old man with a 31 pack‐year smoking history was diagnosed with lung squamous cell carcinoma (T4N1M1c, stage IVB) with left adrenal metastasis. Molecular examination of the tumor genes showed that *EGFR* and *BRAF* were wild type, and *ALK* and *ROS*‐*1* fusion gene rearrangement were not detected. The programmed death‐ligand 1 (PD‐L1) tumor proportion score stained with the PD‐L1 22C3 pharmDx assay was 80%. The patient was treated with four cycles of carboplatin, nab‐paclitaxel, and pembrolizumab, and four cycles of pembrolizumab maintenance. Subsequently, the tumor significantly decreased in size (Figure 1A, B). After four cycles of pembrolizumab maintenance, the patient developed right shoulder and knee arthritis (CTCAE grade 3). Treatment with prednisolone 20 mg was started; however, the arthritis did not improve, and the dose of prednisolone was increased to 70 mg. As 70 mg prednisolone and repeated joint injections of betamethasone reduced arthralgia, the prednisolone dose was tapered to 40 mg.

**FIGURE 2:**
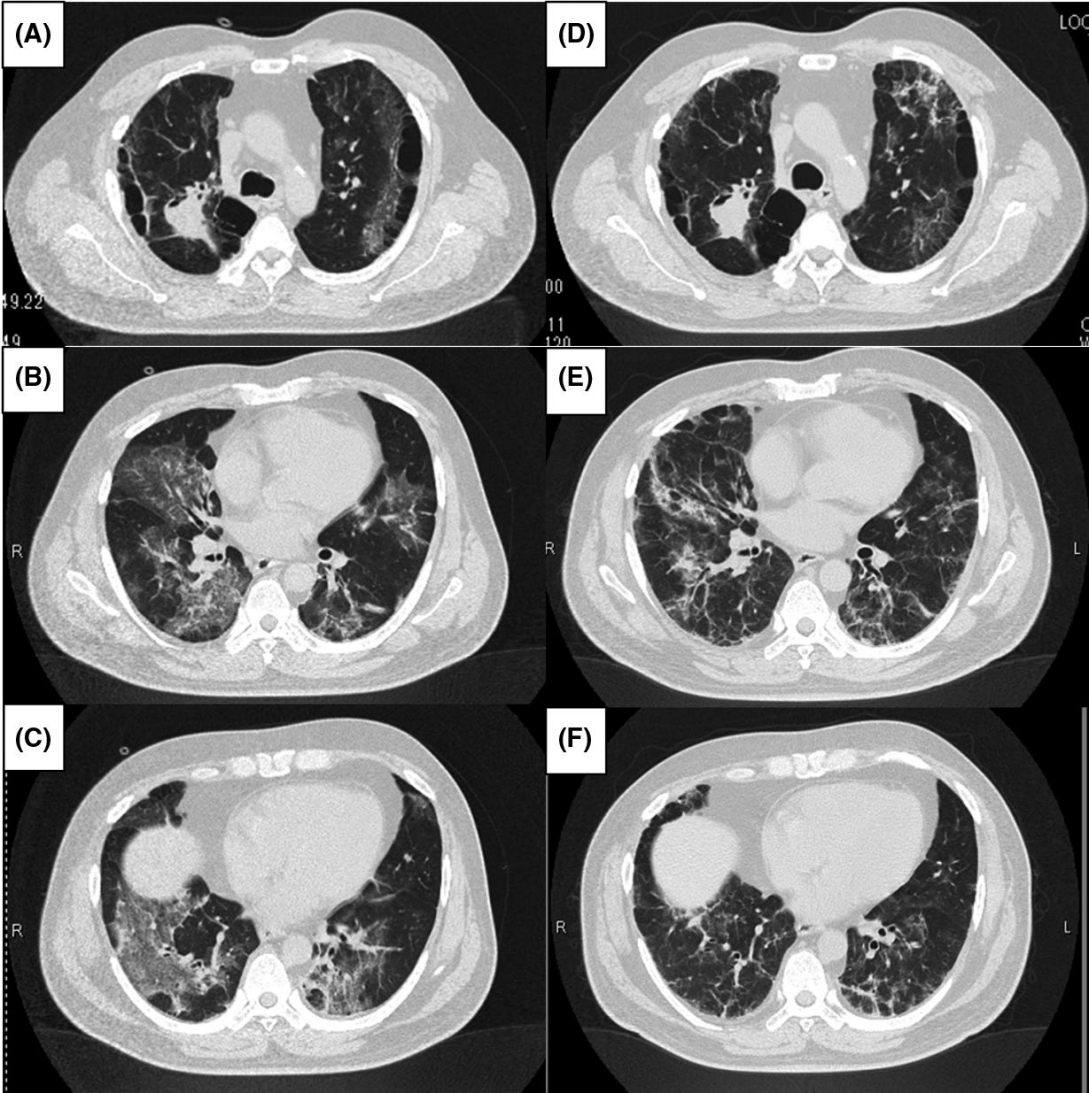
Chest computed tomography showing diffuse ground‐glass opacities in both lungs on the 6th day of COVID‐19 onset (A‐C). Chest computed tomography revealing that ground‐glass opacities in both lungs reduced, but linear and reticular shadows remained on the 29th day of COVID‐19 onset (D‐F)

**FIGURE 3:**
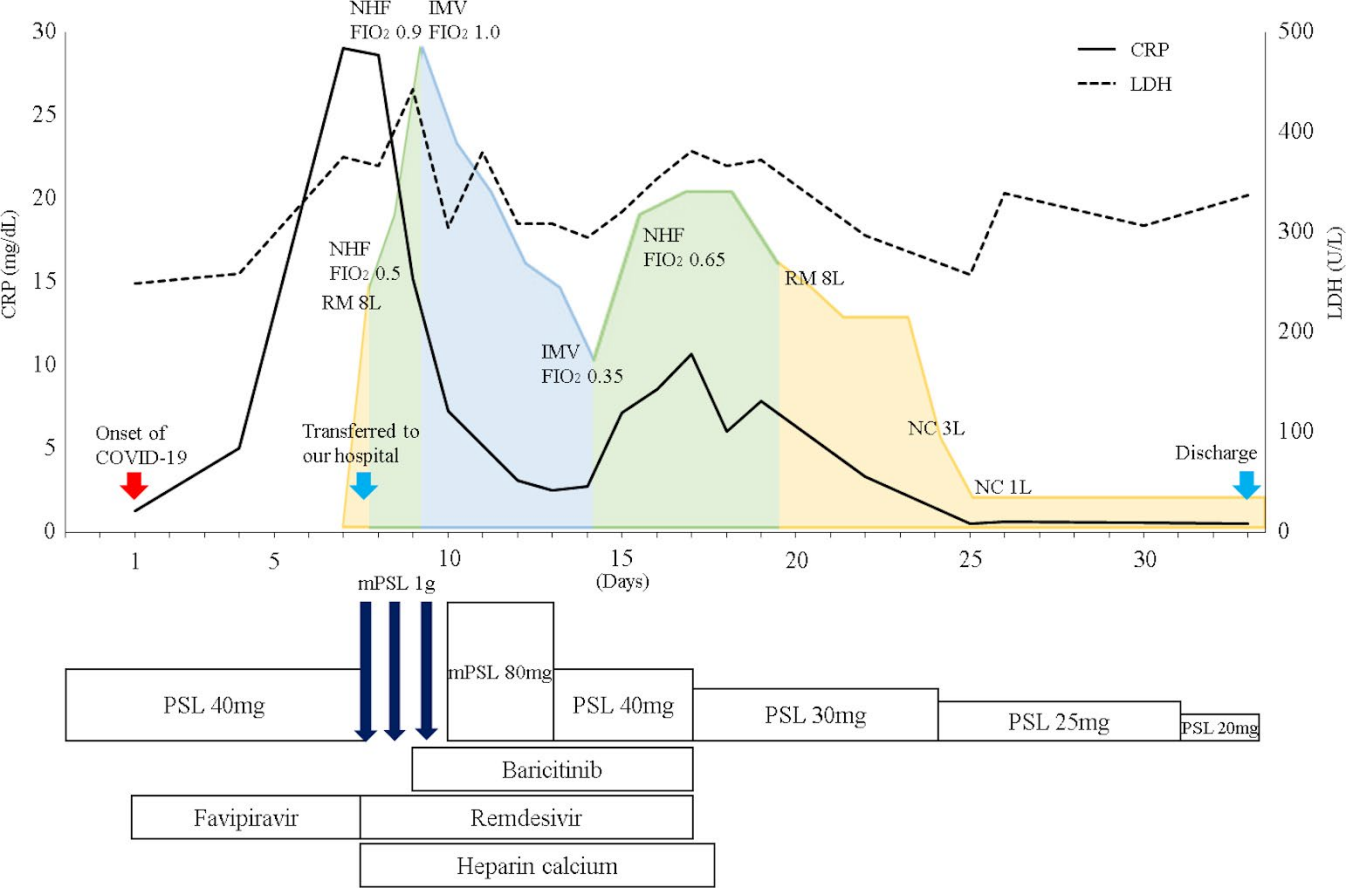
Clinical course. CRP: C‐reactive protein, LDH: lactate dehydrogenase, PSL: prednisolone, mPSL: methylprednisolone, COVID‐19: coronavirus disease 2019, RM: reservoir mask, NHF: nasal high flow, IMV: invasive mechanical ventilation, NC: nasal cannula, and FIO2: fraction of inspiratory oxygen

Thirty days after the administration of prednisolone for immune‐related arthritis (52 days after the last dose of pembrolizumab), the patient developed COVID‐19, as diagnosed by nucleic acid amplification test for severe acute respiratory syndrome coronavirus 2 (SARS‐CoV‐2) using a nasopharyngeal swab. On the 2nd day of COVID‐19 onset, treatment with favipiravir was initiated and prednisolone 40 mg was withheld. On the 7th day of COVID‐19 onset, the patient required oxygen and was transferred to our hospital due to rapid worsening of his respiratory condition within several hours. The patient presented high fever, cough, dyspnea, and arthralgia, and his body mass index was 31.8. His oxygen saturation measured using a pulse oximeter under oxygen inhalation at 8 L/min was 96%. The following blood test results were obtained: white blood cells, 9600/μl; neutrophils, 8611/μl; lymphocytes, 854/μl; hemoglobin, 13.5 g/dl; platelet, 186,000 /μl; D‐dimmer, 2.0 μg/ml; albumin, 3.4 g/dl; creatine kinase, 16 U/L; aspartate aminotransferase, 16 U/L; lactate dehydrogenase (LDH), 375 U/L; creatinine, 0.85 mg/dl; blood urea nitrogen, 14 mg/dl; C‐reactive protein (CRP), 29.07 mg/dl; procalcitonin, 0.31 ng/ml; hemoglobin A1c, 7.0%; and ferritin, 2349 ng/ml. Chest computed tomography (CT) revealed diffuse ground‐glass opacities in both lungs (Figure 2A‐C). Nasal high‐flow oxygen therapy and treatment with 1 g of methylprednisolone, remdesivir, and heparin calcium were started. However, tracheal intubation and invasive mechanical ventilation were required on the 9th day of COVID‐19 onset, and prone ventilation and treatment with 4 mg of baricitinib were started. Thereafter, his respiratory condition gradually improved, and he was extubated on the 14th day of COVID‐19 onset. Chest CT showed that the ground‐glass opacities in both lungs were reduced, but linear and reticular shadows remained (Figure 2D‐F). The patient was discharged on the 33th day of COVID‐19 onset (Figure 3). Prednisolone has been tapered to 10 mg, but there has been no relapse of arthritis. Lung cancer had not progressed at 4 months after cessation of pembrolizumab.

## 3 DISCUSSION

The present case is of a patient with NSCLC who developed severe COVID‐19 while receiving prednisolone 40 mg for refractory pembrolizumab‐induced arthritis. Methylprednisolone pulse and remdesivir could partially suppress the cytokine storm indicated by his serum CRP levels; however, the respiratory condition did not improve, which necessitated invasive mechanical ventilation. After the addition of baricitinib, clinical recovery was achieved. This clinical course suggested that a combination therapy with methylprednisolone, baricitinib, and remdesivir may be effective in rapidly deteriorating patients with critical COVID‐19.

There is no clear consensus about the impact of ICIs on the clinical course of COVID‐19. In the TERAVOLT study, it has been reported that ICIs did not increase the risk of death in thoracic cancer patients with COVID‐19,^3^ and Luo et al^4^. reported that receiving PD‐1 inhibitors did not affect the severity of COVID‐19. Conversely, Robilotti EV et al^5^. reported that ICI administration may increase the risk of severe COVID‐19; there is also a report that ICIs administered within 40 days may increase the risk of death or severe COVID‐19.^2^

Lung pathological findings in a fatal case of COVID‐19 revealed overactivation of cytotoxic CD8+ T cells.^6^ A cytokine storm caused by an excessive immune response is considered responsible for the severe acute respiratory distress syndrome in COVID‐19.^7^ On the other hand, blockade of the PD‐1/PD‐L1 axis induced by ICIs could break the self‐tolerance, reactivating autoimmune CD8+ T cells and leading to irAEs.^8^ There are similarities in the mechanisms of severe COVID and irAEs of ICIs from the perspective of activation of CD8+ T cells. In addition, corticosteroid use ≥20 mg per day equivalent of prednisone was associated with an increased risk of hospitalization of cancer patients with COVID‐19.^5^ Therefore, patients on immunosuppressive agents for the treatment of irAEs of ICIs may be primed for severe COVID‐19, as in the present case.

Corticosteroids have been the mainstay of treatment for COVID‐19 since dexamethasone 6 mg was reported to reduce mortality in hospitalized COVID‐19 patients in the RECOVERY trial.^9^ In this trial, in the dexamethasone group, the incidence of death was lower than that in the usual care group among patients receiving invasive mechanical ventilation (29.3% vs. 41.4%; rate ratio, 0.64; 95% CI, 0.51–0.81) and among those receiving oxygen (23.3% vs. 26.2%; rate ratio, 0.82; 95% CI, 0.72–0.94). However, even in the dexamethasone group, the mortality of severe COVID‐19 was very high and the optimal regimen of corticosteroid and the combination therapy with other agents remains to be investigated. Edalatifard M et al^10^. have reported that a methylprednisolone pulse reduced mortality in severe COVID‐19 patients (not intubated) compared to standard care (5.9% vs. 42.9%; hazard ratio, 0.29; 95% CI, 0.15–0.56), suggesting the efficacy of high‐dose corticosteroid in severe COVID‐19.

Baricitinib, a selective inhibitor of Janus kinase 1 and 2, not only interrupts the passage and intracellular assembly of SARS‐CoV‐2 into the target cells via disruption of AP2‐associated protein kinase 1 signaling, but also inhibits the intracellular signaling pathway of cytokines known to be elevated in severe COVID‐19, including interleukin (IL)‐2, IL‐6, IL‐10, interferon‐γ, and granulocyte‐macrophage colony‐stimulating factor.^11^ In the ACTT2 trial, baricitinib plus remdesivir was shown to shorten the time to clinical recovery in hospitalized COVID‐19 patients compared to remdesivir alone.^12^ In this trial, corticosteroid use rate after enrollment was only 10.9%‐12.9%, and the benefit of adding corticosteroids to baricitinib plus remdesivir was uncertain. One observational study reported possible synergistic effect of a combination therapy with corticosteroid and baricitinib on severe COVID‐19.^13^ Further studies are required to determine the optimal timing of baricitinib administration, in combination with or without corticosteroids.

Rheumatic irAEs are often persistent and can require long‐term treatment with immunosuppressive agents.^14^ Conventional synthetic disease‐modifying antirheumatic drugs (DMARD) should be considered in patients with insufficient response to acceptable dose of corticosteroid or requiring corticosteroid sparing.^15^ The patient in the present case required high‐dose corticosteroid for refractory arthritis, although the corticosteroid dose could be tapered after treatment for COVID‐19. The clinical efficacy and safety of baricitinib, which is a targeted synthetic DMARD, in the context of rheumatic irAEs have yet to be proven.^16^ Intensive immunosuppressive therapy with methylprednisolone pulse and baricitinib for critical COVID‐19 may have led to remission of refractory immune‐related arthritis.

In conclusion, COVID‐19 in cancer patients on immunosuppressive agents for the treatment of irAEs of ICIs can rapidly deteriorate. The combination therapy with methylprednisolone, baricitinib, and remdesivir may be effective for critical COVID‐19, and further clinical trials are warranted.

## CONFLICT OF INTEREST

None declared.

## AUTHOR CONTRIBUTIONS

NO: drafted the manuscript. All authors: have read and approved the final manuscript, and contributed substantially to the revision.

## ETHICAL APPROVAL

The patient provided written informed consent for the publication.
